# Supplementary material for: Effect of X-ray irradiation on ancient DNA in sub-fossil bones – Guidelines for safe X-ray imaging
Source: Sci Rep. 2016 Sep 12;6:32969. doi: 10.1038/srep32969 (PMC5018823; doi:10.1038/srep32969)
Supplement: Supplementary Information [file srep32969-s1.pdf]

Effect of X-ray irradiation on ancient DNA in sub-fossil bones – Guidelines for safe X-ray imaging.

Supplementary Information

Authors

Alexander Immel[1,2*], Adeline Le Cabec[3,4*], Marion Bonazzi[5*], Alexander Herbig[1], Heiko Temming[3], Verena J. Schuenemann[2], Kirsten I. Bos[1], Frauke Langbein[2], Katerina Harvati[6], Anne Bridault[7], Gilbert Pion[8], Marie-Anne Julien[9,10], Oleksandra Krotova[11], Nicholas J. Conard[12], Susanne C. Münzel[13], Dorothée G. Drucker[14], Bence Viola[15,3], Jean-Jacques Hublin[3], Paul Tafforeau[4#] and Johannes Krause[1,2,12#]

* These authors contributed equally, # corresponding author for X-ray imaging and dosimetry: paul.tafforeau@esrf.fr, # corresponding author for aDNA analysis: krause@shh.mpg.de

Affiliations

[1]Department of Archaeogenetics, Max Planck Institute for the Science of Human History, Jena, Germany;

[2]Institute for Archaeological Sciences, Archaeo- and Palaeogenetics, University of Tübingen, Tübingen, Germany;

[3]Department of Human Evolution, Max Planck Institute for Evolutionary Anthropology, Leipzig, Germany;

[4]European Synchrotron Radiation Facility, Grenoble, France;

[5]Institute of Clinical Molecular Biology, Kiel University, Kiel, Germany;

[6]Senckenberg Center for Human Evolution and Palaeoecology, Palaeoanthropology, University of Tübingen, Tübingen, Germany;

[7]CNRS UMR 7041 ArScAn, Equipe Archéologies environnementales, F-92023 Nanterre Cedex, France;

[8]Association départementale pour la recherche archéologique en Savoie, F-73230 Saint-Alban-Leysse, France;

[9]Centre for the Archaeology of Human Origins, Archaeology Department, University of Southampton, Southampton SO17 1BF, United Kingdom;

[10]Unité Histoire naturelle de l'Homme préhistorique (UMR 7194), Sorbonne Universités, Muséum national d'Histoire naturelle, CNRS, Paris, France;

[11]Department of Stone Age, Institute of Archaeology, National Ukrainian Academy of Science, Kiev, Ukraine;

[12]Senckenberg Center for Human Evolution and Palaeoenvironment, University of Tübingen, Tübingen, Germany;

[13]Institute for Archaeological Sciences, Archaeozoology, University of Tübingen, Tübingen, Germany;

[14]Department of Geosciences, Palaeobiology, University of Tübingen, Tübingen, Germany;

[15]Department of Anthropology, University of Toronto, Toronto, Canada;

Supplementary Note 1 – X-ray imaging and dose deposition

The first critical aspect to assess X-ray dose is linked to the voxel size of the scan (often presented as resolution, even if this term is not really adapted). Synchrotron µCT is using parallel beam geometry; hence the resolution and magnification are obtained thanks to the X-ray detector. In most of the cases, it is an indirect detector based on a scintillator screen (mostly single crystals for high quality phase contrast imaging) coupled to a CCD or CMOS camera

through an optical device (e.g. microscope, photographic objectives, optical taper). For a constant X-ray spectrum, the X-ray dose necessary to perform a scan with a given voxel size depends mostly on the detector properties. By opposition, in conventional sources, the magnification effect is obtained only thanks to the conical geometry of the X-ray source, by displacing the sample along the X-ray cone. In this case, for constant X-ray spectrum, the dose necessary for a scan of a given voxel size depends on the distance between the source and the detector. This major difference between synchrotron and conventional X-ray source makes possible to derive X-ray dose relatively easily for conventional sources from calibration points by geometric calculation, whereas it is necessary to test all the different detectors combinations (scintillator/optic/sensor) for the synchrotron configurations.

The second critical aspect for X-ray dose is linked to the X-ray spectrum. Low energy X-rays are more easily absorbed by the samples than high energy X-rays. Nevertheless, low energy X-rays can also bring higher contrast level than high energy ones. Tomography requires that a sufficient amount of X-rays goes through the sample (typically the lowest transmission has to be above 10%, but often results are better for minimum transmission above 20%). In case of broad X-ray spectrum, the low energies can be completely absorbed by the sample when higher energies can go through (Supplementary Fig. 1). This very well-known effect is called beam hardening, which is leading to typical artifacts when scanning with such broad spectrum. Nowadays, very efficient algorithms can correct most of these artifacts [1], but it remains that the low energy photons are depositing a large part of the dose as they can be totally absorbed. Adapted filtering of the source spectrum using adapted metallic filter allows removing the lowest energies in order that the X-rays used to scan the specimen are really useful to obtain the data.

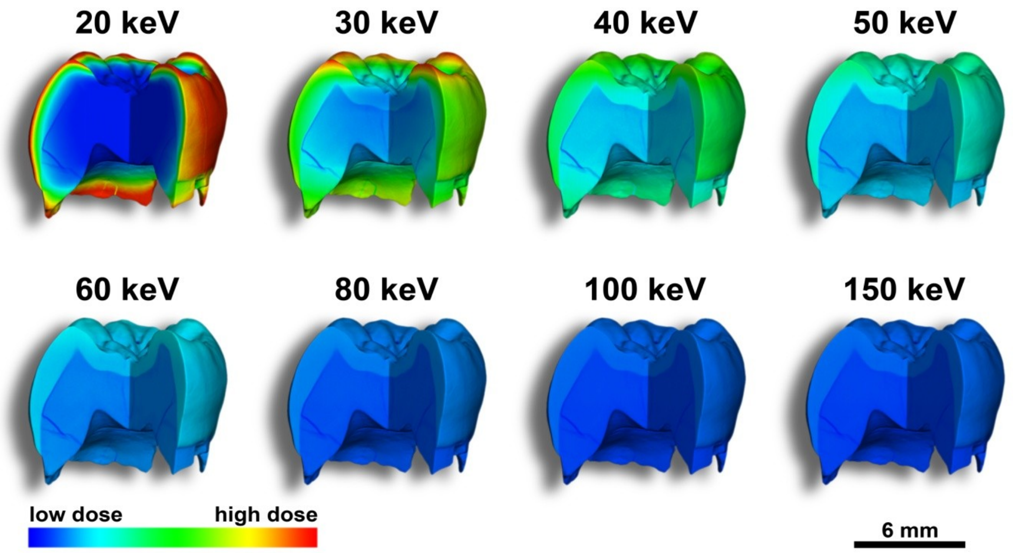

Supplementary Figure 1. 3D simulation of dose deposition pattern in a fossil molar depending on the photons energy (lower first molar of the Engis 2 Neandertal child from Belgium, scan was originally published in Smith *et al*. 2010[2], data deposited on the ESRF public database http://paleo.esrf.eu). Calculations are based on a constant amount of photons for the different energies, i.e. not for constant surface dose. Low energies are completely stopped in the superficial layers of the sample and will then deposit high dose level in surface and subsurface, when high energies go through the sample in a more uniform way.

When performing X-ray tomography, it is therefore important to balance all these effects to find the optimal configurations allowing the narrower X-ray spectrum, with energy high enough to reach sufficient transmission through

the sample without going to too high energy that would tend to reduce contrast.

Synchrotrons are well known to allow imaging using monochromatic beam, that remove *de facto* the beam hardening effect[1,3]. Nevertheless, narrow polychromatic beam can bring results very close, without visible beam hardening, while allowing less ring artifacts (better beam profile and stability), and faster scans.

Since 2011, all the fossil specimens scanned on the beamline ID19 at the ESRF are imaged using these high quality direct "pink" (meaning narrow spectrum polychromatic) beams[4-6]. It has to be noted that the use of high quality pink beam or of monochromatic beam does not really have an impact on the delivered dose since in both cases the average energies are similar as well as the dynamic level on the detector.

Imaging of fossils with synchrotrons also implies in nearly all the cases the use of propagation phase contrast. This technique can be up to 1000 times more sensitive to small density differences than X-ray absorption used in conventional systems, and is often giving better results with energies higher than those used for absorption. Phase contrast is nowadays the most important reason why using synchrotron sources to image fossils, especially for observation of small structures such as incremental lines in teeth or bones microstructures. It is also the key to reduce the X-ray dose for sub-fossils by using as much as possible the high sensitivity given by this approach. All the results presented in the present study for low dose synchrotron imaging are based on propagation phase contrast and would not be relevant for pure absorption imaging.

Classical sub-μm resolution configurations could even reach the level of total destruction of aDNA, but these configurations are restricted to small irradiated volumes (Supplementary Fig. 2) thanks to the precise beam collimation of synchrotron sources, i.e. the possibility to adapt the beam size to the field of view using absorbing slits systems.

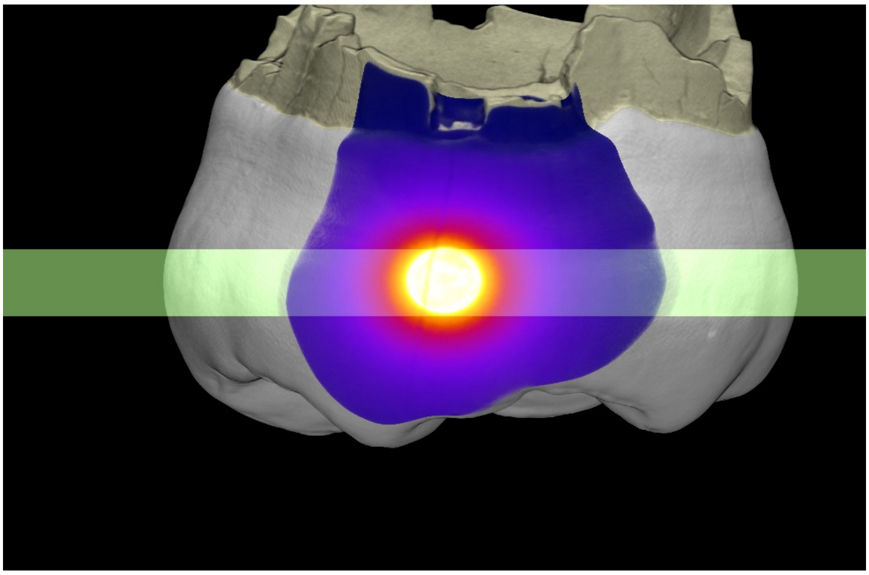

Supplementary Figure 2. Typical 3D dose deposition pattern of a sub-μm resolution scan for enamel microstructure as performed at the ESRF (3D simulation performed on the same lower first molar of the Engis 2 Neandertal child as in Supplementary Fig. 1). Only the central yellow part can reach the high dose level reported in the present study, the dose rapidly decreases for all the other parts crossed by the beam during the scan depending on the distance to the imaged part. Beam scattering (not simulated here) will also contribute to general dose level, but high resolution dosimetry experiments shows that it remains far less important than the dose deposition due to the direct beam, and its contribution decreases very rapidly with the distance to the direct beam (negligible after typically 200 μm in the geometry presented here).

Low resolution scans (voxel size larger than 20 μm, implying full irradiation of the specimens) were typically in the

safe zone even with classical configurations, but the new configurations are well below the detection limit of 200 Gy. The most detrimental configurations with the classical synchrotron scans were in the 5 µm range, where large areas were scanned while having substantial level of dose (typically complete teeth for dental development), but these scans would not have really endangered aDNA studies, except in case of multiple scans. The highest efforts for dose reduction were applied in this resolution range, as it was potentially the most dangerous one, and as it is a critical one for virtual dental and bones palaeohistology. The new configurations implemented on ID19 are now well below the detection limit. Nowadays, only the sub-µm resolution scans typically used to observe enamel microstructures can still reach dose level that could have limited consequences on aDNA, but due to the small beam size, the concerned areas are very limited (typically small cylinders of 4*2 mm), and concern mostly enamel, where no sampling for aDNA would be done anyway. All in all, the complete set of configurations available at the ESRF for scanning of sub-fossils can be considered as safe for future aDNA studies, as long as good care is taken to perform the experiments (especially avoiding multiple scans whenever they are not necessary). Further efforts are ongoing to decrease dose for the sub-µm setup by further factor 2 to 3, and setups for voxel sizes larger than 10 µm by factor 2 to 10.

In the case of conventional X-ray imaging, it has to be noted that the high surface dose obtained for scans without any filters are due mostly to low energy X-rays. This effect was especially visible with the skyscan1273 scanner for which dose rates without filters were really higher than expected after the experiment on the BIR scanner. Even if the cause of this higher dose is not really clear, it appears to be due to the low energy part of the spectrum as even a thin aluminium filter can completely remove it. Hence, it could lead to substantial aDNA degradation in the sub-surface of a specimen, but not in depth, because the specimen itself would act as a filter and stop these low energy X-rays.

Supplementary Note 2 – X-ray spectrums used for irradiation and imaging experiments

Experiment 1, extreme irradiation

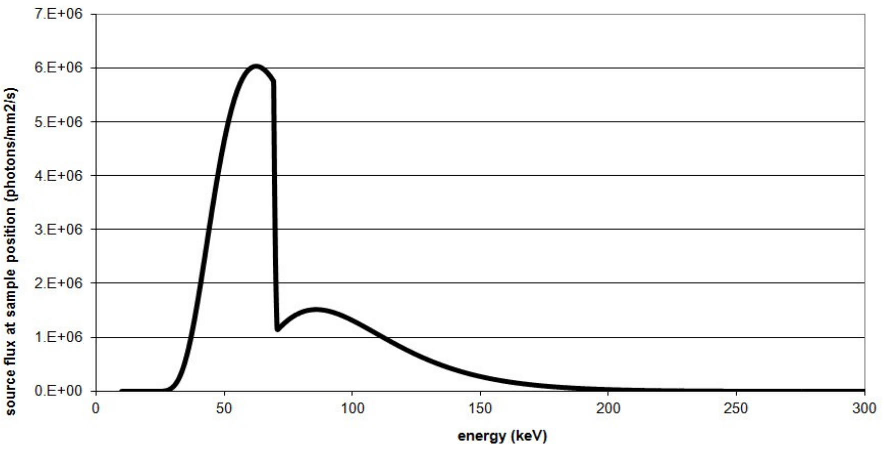

Supplementary Figure 3. X-ray spectrum used for the extreme irradiation experiment on the beamline BM05 using the white beam produced by the 0.85 tesla bending magnet filtered with 3 mm of aluminium, 0.1 mm of copper and 0.1 mm of tungsten. Sample at 50 m from the source. Dose rate of 83.41 Gy/s.

Experiment 2, exposure time series

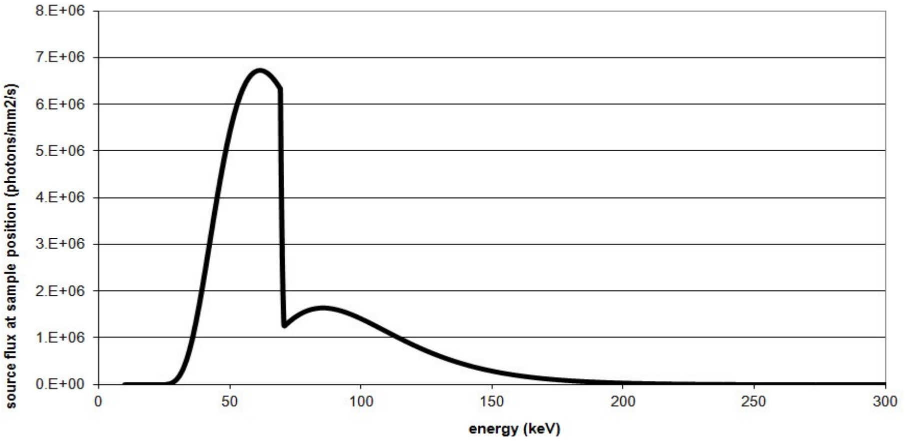

Supplementary Figure 4. X-ray spectrum used for the time series irradiation experiments on the beamline BM05 using the white beam produced by the 0.85 tesla bending magnet filtered with 1.5 mm of aluminium, 0.1 mm of copper and 0.1 mm of tungsten. Sample at 50 m from the source. Dose rate of 93.72 Gy/s.

Experiment 3, synchrotron high quality imaging tomography

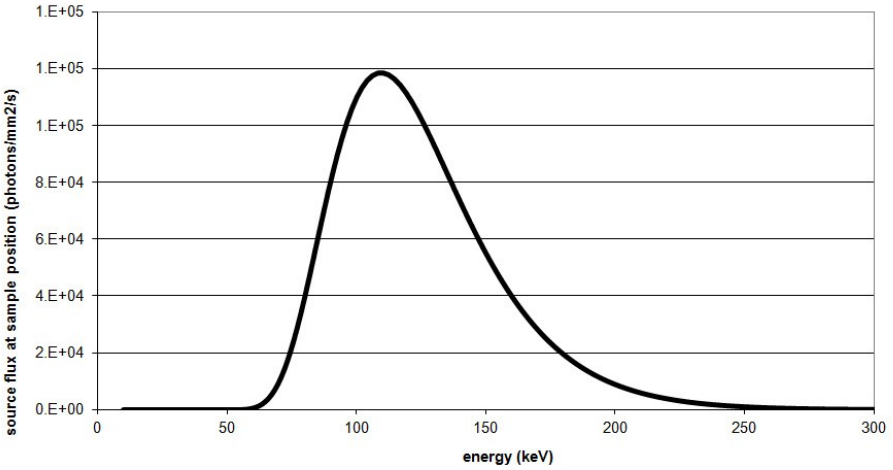

Supplementary Figure 5. X-ray spectrum used for the high quality imaging synchrotron tomography experiment on the beamline BM05 using the white beam produced by the 0.85 tesla bending magnet filtered with 23 mm of aluminium and 6 mm of copper. Sample at 50 m from the source. Dose rate of 1.61 Gy/s.

Supplementary Note 3 – X-ray device for luggage inspection in airports

The following information results from a correspondence with Dr. Andreas Frank (Director Technology Low Energy Systems Hardware), Dr. Arno Folkerts and Dr. Christian Rauth (Radiation Safety Officer) from Smiths Heimann GmbH, Wiesbaden, Germany. Hyperlinks in the following text will lead the reader to the website of the company as well as further technical details on the device (pdf to download).

Smiths Detection is a company that produces X-ray scanning devices for luggage inspection in airports. HiScan 6040 a-TiX is the current model in use by the security staff in most German airports (including Leipzig and Frankfurt) and many international airports as well.

The scanning device is equipped with four X-ray sources, each emitting a thin fan-beam which penetrates the luggage during security inspection. The luggage progresses through the tunnel of the device at a constant speed of 20 cm/s. The total exposure time is about 20 ms (5 ms for each of the 4 X-ray sources). The machines are configured in a way that the dose is always delivered in the same way, and this cannot be changed by the security staff at the airport. This results in a total delivered dose of 10 to 12 µGy per inspection process.

Smith Detection uses several dosimetry protocols for cross-validating their results, and although not directly and strictly obtained the same way as our values on conventional and synchrotron CT were acquired, the values provided above remain comparable, since being in the range of 10 µGy.

Occasionally, the security staff may repeat the procedure if the image quality is not satisfying enough, although this is not standard procedure in most airports. Further inspections would rather be conducted (e.g., opening and searching the luggage), possibly involving other technologies (e.g., trace detection).

Some airports in Europe still use older models of devices for luggage inspection (e.g., HiScan 6040i or HiScan 6046si) which only have one X-ray source, and for which the total delivered dose per inspection is 1.5 to 2.5 µGy (for 5 ms exposure time).

Supplementary References

1 Olejniczak, A. J., Tafforeau, P., Smith, T. M., Temming, H. & Hublin, J. J. Technical note: compatibility of microtomographic imaging systems for dental measurements. *American journal of physical anthropology* **134**, 130-134, doi:10.1002/ajpa.20615 (2007).

2 Smith, T. M. *et al.* Dental evidence for ontogenetic differences between modern humans and Neanderthals. *Proceedings of the National Academy of Sciences of the United States of America* **107**, 20923-20928, doi:10.1073/pnas.1010906107 (2010).

3 Tafforeau, P. *et al.* Applications of X-ray synchrotron microtomography for non-destructive 3D studies of paleontological specimens. *Applied Physics A* **83**, 195-202 (2006).

4 Le Cabec, A., Tang, N. & Tafforeau, P. Accessing developmental information of fossil hominin teeth using new synchrotron microtomography-based visualization techniques of dental surfaces and interfaces. *PloS one* **10**, e0123019, doi:10.1371/journal.pone.0123019 (2015).

5 Sanchez, S., Ahlberg, P. E., Trinajstic, K. M., Mirone, A. & Tafforeau, P. Three-dimensional synchrotron virtual paleohistology: a new insight into the world of fossil bone microstructures. *Microscopy and microanalysis: the official journal of Microscopy Society of America, Microbeam Analysis Society, Microscopical Society of Canada* **18**, 1095-1105, doi:10.1017/s1431927612001079 (2012).

6 Smith, T. M. *et al.* Dental ontogeny in pliocene and early pleistocene hominins. *PloS one* **10**, e0118118, doi:10.1371/journal.pone.0118118 (2015).

Supplementary Table 2 - Scanned Specimens at MPI-EVA and delivered X-ray Doses

Site	Taxon	Specimen ID	bone/tooth	ESTIMATED DOSE	Ontoitem	scan ID	Scanner	kV	µA	filters	total scanning time	nb of scans	nb of projections	frame averaging	exposure time [ms]	rotation step (deg)	Dis-tance [ob-ject] to source [mm]	geometry	Pixel size [um]
Baigara	H. sapiens	Baigara 1	bone	**0.9 Gy**	TALL	10001241/000/001	BIR SN001 ACTIS5	130	110	0.25 mm Brass	02:13:20	3	2500	3	333.3	0.144	330.0	360	63.728
Chagyrskaya	Neandertal	Chagyrskaya 2	bone	**9.7 Gy**	C1	20003197/000/001	Skyscan 1172	100	100	0.5 mm Al + 0.04 mm Cu	04:26:10	3	3000	2	2655	0.120	260.1	360	17.3974
Chagyrskaya	Neandertal	Chagyrskaya – SP3393	bone	**18.7 Gy**	HP	20003919/000/001	SkyScan1173	100	62	Al 1.0 mm	03:22:40	1	2400	4	991	0.150	85.9	360	11.8304
Chagyrskaya	Neandertal	Chagyrskaya – SP3394	bone	**8.3 Gy**	ULNL | ULNR	20003920/000/001	SkyScan1173	100	62	Al 1.0 mm	03:22:26	2	2400	4	991	0.150	182.2	360	25.09851
Denisova	Denisovan	Denisova 2 - SP3276	tooth	**25.8 Gy**	LLDM2	20003694/000/001	SkyScan1173	100	62	Al 1.0 mm	03:23:26	1	2400	4	991	0.150	72.9	360	10.03552
Denisova	?	Denisova 3	?	**15.2 Gy**	?	?	skyscan 1172	100	100	Al 0.5 mm	02:57:00	1	3000	2	885	0.120	127.3	360	6.8
Denisova	Denisovan	Denisova 4	tooth	**1.5 Gy**	ULM3	10000796/002/001	BIR SN001 ACTIS5	130	100	0.25mm Brass	?	?	2500	2	333.3	0.144	110.0	?	20.014
Denisova	Neandertal	Denisova 5	bone	**9.9 Gy**	FP4or5P	20003531/000/001	Skyscan 1172	100	100	0.5 mm Al + 0.04 mm Cu	03:31:50	2	2400	2	1770	0.150	187.6	360	15.4595
Denisova	?	Denisova 8	tooth	**5.4 Gy**	molar	10001219/000/004	BIR SN001 ACTIS5	130	110	0.25 mm Brass	02:46:40	4	2500	3	333.3	0.144	165.0	360	30.065
Ust'-Ishim	H. sapiens	Ust'-Ishim 1	bone	**0.3 Gy**	FEML	10001598/000/001	BIR SN001 ACTIS5	130	100	0.50 mm Brass	00:56:40	9	2500	2	333.3	0.144	500.0	360	91.236
Vindija	Neandertal	Vi 33.16 (Vi 80)	bone	**0.4 Gy**	TIB?	10000096/001/001	BIR SN001 ACTIS5	130	100	0.25 mm Brass	00:55:33	4	1250	2	333.3	0.288	300.0	360	54.6
